# Response to “How prepared is Africa to face COVID-19?” by Wadoum and Clarke

**DOI:** 10.11604/pamj.supp.2020.35.2.23829

**Published:** 2020-06-03

**Authors:** John Koku Awoonor-Williams, James Franklin Phillips, Stephen Patrick Kachur, Elizabeth Fletcher Jackson, Rachel Tara Moresky, Dennis Chirawurah

**Affiliations:** 1 Director, Policy Planning Monitoring and Evaluation Division, Ghana Health Service, Accra, Ghana; 2 Mailman School of Public Health, Columbia University, New York, USA; 3 University of Development Studies, Tamale, Ghana

**Keywords:** Africa, COVID-19, health systems, social norms

## Abstract

A recent commentary published in this journal correctly notes the important challenges that must be addressed to mitigate the effects of the COVID-19 pandemic in Africa. While we agree with the basic assumptions and arguments of their essay, we argue that common social institutional norms in most rural settings could be marshalled for organizing preventive measures.

## Commentary

A recent commentary in the Pan African Medical Journal (PAMJ) entitled “How prepared is Africa to face COVID-19?” by Raoul Emeric Guetiya Wadoum and, Andrew Clarke [1], outlined events associated with the onset of the COVID-19 pandemic with an insightful summary of the recent history of African Union (AU) organizational preparations for public health emergencies. We welcome this commentary and agree with its major themes. However, there are elements of the commentary that merit further reflection. The authors state *“As the world mobilizes to support the WHO declaration of COVID-19 a Public Health Emergency, and subsequently struggles to manage the rapid emergence of the infection, scientists and public health experts are raising a serious alarm about the catastrophic effects that an outbreak in Africa could have, given that public health systems throughout Africa are weak and most of the continent lacks the global health security capabilities and social protection infrastructure necessary to adequately respond and manage outbreaks and the cascade of subsequent effects on society, in particular the most vulnerable sections.”* The commentary is correct in expressing concern about the potential societal impact of the pandemic, given the fundamental resource constraints that have constrained health systems responses to health crises in the past. The commentary noted: *…as we witness the rapid spread of SARS-CoV-2 across increasingly diverse and large parts of the world’s population, within countries with greater resource and organizational capacity to draw upon than most in Africa, and despite radical restrictions on population movement, we can only brace ourselves and demand extensive planning based on evidence and pre-mobilization of the resource.* Our concern, however, is that these appropriate calls for action inadequately acknowledge social resources that exist in the region and their possible contribution to pandemic responses. Progress in the prevention and treatment of childhood illness has been impressive in recent decades, to a significant degree due to successful social mobilization for primary health care development [2]. Social protection infrastructure may be weak in the Western sense of social bureaucratic capacity. However, if the strategic implications of social organization and links of tradition with institutional capacity in Africa are considered, the organizational traditions of family, extended family, kindred group, and community are far more developed and robust than is the case in Europe, North America, or the regions of Asia. The commentary notes, furthermore, that *“…given the fragility of our health systems, it is hard to foresee how any level of a surge in demand for high-intensity health care will be accommodated.”*

Understanding how social organization can enable community engagement activities has been critical to the expansion of community-based primary health care services throughout Africa [3]. In most of rural Africa, initiatives that require volunteerism, collective action, and social networking are more feasible to implement than has been the case elsewhere. The widespread deployment of community health workers has been critical to primary health care development in the region through initiatives that require volunteerism, collective action, and social networking are more feasible to implement than has been the case elsewhere. Multiple studies attest to the capacity of CHW to be entrusted with complex social outreach activities that aim to change health behavior [4]. This capability facilitates implementation of the type of interventions that the commentary appropriately promotes [5]. Community health workers can serve a vital role in ensuring that essential preventive and curative health services for other conditions are sustained without catastrophic interruption [6]. The phrase “fragility of our health systems” does not apply to African social systems. If epidemic control measures are grounded in social institutions and developed and tested in Africa, with appropriate adaptations, then effective means of controlling pandemics may be feasible to implement at low cost. Disease control strategies for implementing case identification, isolation, contact tracing, and quarantine will be facilitated by the social context rather than constrained by a lack of high intensity health care [7-9]. Finally, these strategies have been effectively integrated with the World Health Organization (WHO)´s Early Warning Alert and Response Network (EWARN) for the early detection of epidemic-prone diseases. Successful applications of this system rely on partnerships which engage communities and CHWs like in Cyclone Ida which struck Mozambique in March. Though previous community engagement, the Mozambican Ministry of Health was able to galvanize 1200 volunteers to support cholera vaccine campaign [10]. While we agree with the view, expressed in this commentary, that concerted action is needed, prospects for doing so will be enhanced by building initiatives that marshal the organizational opportunities that existing African social institutions provide.

## Competing interests

The author declares no competing interests.
